# Transcriptional Regulation of Latent Feline Immunodeficiency Virus in Peripheral CD4+ T-lymphocytes 

**DOI:** 10.3390/v4050878

**Published:** 2012-05-23

**Authors:** Samantha J. McDonnel, Ellen E. Sparger, Paul A. Luciw, Brian G. Murphy

**Affiliations:** 1 Department of Pathology, Microbiology & Immunology, School of Veterinary Medicine, University of California, Davis, 4206 Vet Med 3A, Davis, CA 95616, USA; Email: bmurphy@ucdavis.edu; 2 Department of Medicine and Epidemiology, School of Veterinary Medicine, University of California, 3115 Tupper Hall, Davis, CA 95616, USA; Email: eesparger@ucdavis.edu; 3 Department of Pathology and Laboratory Medicine, Center for Comparative Medicine, University of California, Davis, County Road 98 and Hutchison Drive, Davis, CA 95616, USA; Email: paluciw@ucdavis.edu

**Keywords:** FIV, latency, lentivirus, chromatin, transcription, CD4 T-cells

## Abstract

Feline immunodeficiency virus (FIV), the lentivirus of domestic cats responsible for feline AIDS, establishes a latent infection in peripheral blood CD4+ T-cells approximately eight months after experimental inoculation. In this study, cats experimentally infected with the FIV-C strain in the asymptomatic phase demonstrated an estimated viral load of 1 infected cell per approximately 10^3^ CD4+ T-cells, with about 1 copy of viral DNA per cell. Approximately 1 in 10 proviral copies was capable of transcription in the asymptomatic phase. The latent FIV proviral promoter was associated with deacetylated, methylated histones, which is consistent with a condensed chromatin structure. In contrast, the transcriptionally active FIV promoter was associated with histone acetylation and demethylation. In addition, RNA polymerase II appeared to be paused on the latent viral promoter, and short promoter-proximal transcripts were detected. Our findings for the FIV promoter in infected cats are similar to results obtained in studies of human immunodeficiency virus (HIV)-1 latent proviruses in cell culture *in vitro* studies. Thus, the FIV/cat model may offer insights into *in vivo* mechanisms of HIV latency and provides a unique opportunity to test novel therapeutic interventions aimed at eradicating latent virus.

## 1. Introduction

Feline immunodeficiency virus (FIV) is a lentivirus of domestic cats similar to human immunodeficiency virus (HIV) in genome structure and immunopathogenesis [1,2], and has been utilized as the only naturally occurring animal model of immunodeficiency for HIV infection in people [3]. Studies in our laboratory demonstrated that domestic cats experimentally infected with FIV enter a state of latency in peripheral blood CD4+ T-cells with concurrent undetectable plasma viremia approximately eight months after inoculation. Thus, FIV infection of its natural host can provide critical insight into generalizable mechanisms of lentiviral latency and allow for the investigation of novel therapeutics targeting latent viral reservoirs.

## 2. Viral Load

**Figure 1:**
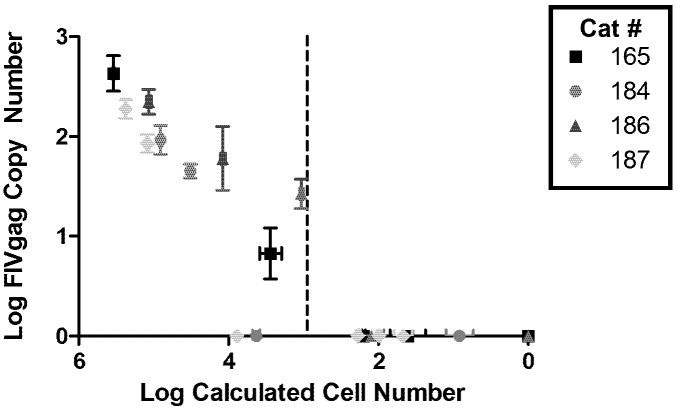
Quantification of feline immunodeficiency virus (FIV) proviral load in CD4+ T-cells. Log FIV *gag* copy number is plotted against the log of calculated cell number (based on cellular IL2 gene copies divided by 2) for 4 chronically FIV-infected cats (28–32 months post infection). Dashed vertical line represents the average cell number at which the FIV signal falls below detection level (~10^3^ cells). Error bars represent the standard deviation of quadruplicate qPCR measurements.

To further characterize the latent FIV reservoir during chronic infection, viral DNA loads were quantified in peripheral CD4+ T-cells using limiting dilution real-time PCR. CD4+ T-lymphocytes were isolated from the peripheral blood of four cats experimentally infected with FIV-Cpgmr [4] in the asymptomatic phase (28–32 months post inoculation) using dual magnetic column separation [5]; flow cytometry showed that the enriched cell population was greater than 97% CD4+ T-cells. Analyses were performed on total CD4+ T cells; within the limitations of the assay, further definition of CD4+ T cell sub-populations was not possible. Approximately 10^6^ CD4+ T-cells from each cat were serially diluted ten-fold down to 10^0^ cells for subsequent DNA isolation (DNA Micro Kit, Qiagen). DNA copy number was measured by real-time PCR for the capsid open reading frame of FIV *gag* and for a portion of the feline interleukin (IL)-2 gene (Figure 1), as previously described [5]. An average of 0.0013 (standard deviation ±0.0005) FIV copies per cellular equivalents (copies of IL-2 divided by 2) were detected and equated to one FIV proviral *gag* copy per 770 (~10^3^) CD4+ cellular equivalents. Thus, the amount of FIV proviral genome copies was ~3 logs less than cellular equivalents with FIV *gag* signal detectable at 10^4 ^cells but lost at 10^3^ cells. Based on the limit of detection of ~10^1^ copies FIV *gag* DNA for this assay, we can conclude that on average there was only one provirus per infected cell, and one infected cell per 10^3^ CD4+ T-cells. In other words, in 10^3^ cells we would expect on average one copy of FIV *gag* as long as there is only one copy of FIV per infected cell; but given that one copy of *gag* is below our limit of detection, 10^4^ cells would be the necessary to detect FIV DNA (which we found to be the case). The FIV proviral DNA load in peripheral CD4+ T-cells was similar to that of HIV-infected humans in the asymptomatic phase [6,7]. It has been suggested that the FIV-Cpgmr isolate is highly virulent [8]; thus, it is unknown whether the proviral loads found here would be similar for other strains of FIV. Though we have previously demonstrated a lack of 2-LTR circle junctions in cells latently infected with FIV [5], it should be noted that the PCR assay used here does not discriminate between integrated and unintegrated viral species such that these figures may provide an over-estimation of proviral load.

To determine the amount of replication competent virus in this reservoir, CD4+ T-cells were isolated from two of the four FIV-infected cats (34–37 months post inoculation) as above, serially diluted from 10^6^ down to 10^2^ cells and cocultured with specific pathogen-free (SPF) feline peripheral blood mononuclear cells (PBMC) for 21 days in mitogen (phorbol myristate acetate and concanavalin A)-containing medium. Supernatant samples were removed on culture days 7, 14, and 21 for DNA and RNA isolation (AllPrep DNA/RNA mini kit, Qiagen), which were assayed for FIV *gag* RNA and 2-LTR circle junctions *via* real-time PCR [5]. On culture days 7 and 21, clarified supernatants were transferred to cultures of fresh SPF feline PBMC and assayed for FIV *gag* DNA after 7 days of incubation. CD4+ T-cells from both cats were initially negative for both FIV RNA and 2-LTR circle junctions (day 0). After 21 days in culture, cultures of as little as 10^4^ CD4+ T-cells from FIV-infected cats were positive for FIV RNA, while cultures containing 10^5^ cells demonstrated infectious supernatants and 2-LTR circle junctions (Table 1). Given that there is approximately one provirus in every 10^3^ CD4+ T-cells (above), these data indicate that approximately 1 in every 10 proviruses is capable of transcription, but, similar to HIV [6,9], only about 1 in 100 proviruses is fully replication competent. Limited sensitivity of the supernatant transfer assay, or viral replication restricted to cell-to-cell spread, may account for the differences among levels of viral RNA, 2-LTR circle junctions, and infectious supernatant observed at the 10^4^ and 10^5^ CD4+ T-cell dilutions.

**Table 1:** Limiting dilution CD4+ T-cells from 2 feline immunodeficiency virus (FIV)-infected cats cocultured with specific pathogen-free (SPF) feline peripheral blood mononuclear cells (PBMC).

Cat Number	Starting CD4+ T-cells	FIV gag RNA			2-LTR Circle Junction			Infectious Supernatant	
		d7	d14	d21	d7	d14	d21	d7	d21
165	10^6^	+	+	+	+	+	+	+	+
	10^5^	+	+	+	+	+	+	-	+
	10^4^	+	+	+	+	-	+	-	-
	10^3^	-	-	-	-	-	-	-	-
	10^2^	-	-	-	-	-	-	-	-
186	10^6^	+	+	+	+	+	+	+	+
	10^5^	+	+	+	+	+	+	-	+
	10^4^	-	-	+	-	-	-	-	-
	10^3^	-	-	-	-	-	-	-	-
	10^2^	-	-	-	-	-	-	-	-

Data are derived from triplicate real-time PCR reactions on cellular DNA (2-LTR CJ, infectious supernatant) or cDNA (FIV *gag* RNA) on day 7 (d7), day 14 (d14), or day 21 (d21). Infectious supernatant was detected by the presence of FIV DNA in SPF PBMC cultured for 7 days with clarified supernatants.

## 3. Chromatin Status

**Figure 2:**
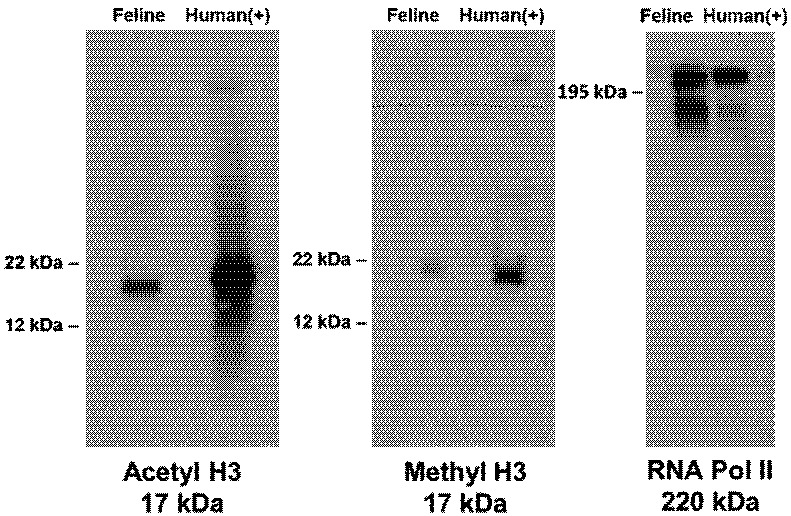
Validation of three antibodies for use in feline ChIP assays. Feline PBMC protein lysate (left) is compared to a positive control of human fetal kidney (293) cell protein lysate (right). Appropriate-sized bands are evident in Western blots incubated with primary anti-acetylated histone H3 (17 kDa), anti-methylated histone H3 (17 kDa), and anti-RNA Polymerase II (220 kDa) antibodies in both lanes.

We hypothesized that histone proteins physically associated with the FIV proviral promoter in latently-infected CD4+ T-cells would possess epigenetic modifications associated with a condensed chromatin structure [10]. PBMC and CD4+ T-cells were isolated from chronically infected cats as described above, and confirmed to be latently infected by detection of FIV *gag* DNA in the absence of detectable FIV *gag* RNA or 2-LTR circle junction DNA *via* real-time PCR [5]. Chromatin immunoprecipitation (ChIP) was performed on these cells by methods described elsewhere [11], using polyclonal rabbit IgG antibodies against either acetylated histone H3 (H3K9,14ac, Millipore), methylated histone H3 (H3K27me3, Millipore), or RNA polymerase II (Abcam). All antibodies were validated for detection of feline proteins in feline PBMC by Western blot (Figure 2), with polyclonal rabbit IgG (Millipore) used as an isotype control. Following ChIP, DNA was assayed by real-time PCR for the 5’ FIV LTR, with a forward primer in U3/R and reverse primer in the *gag* leader sequence, and concurrently for the feline housekeeping gene GAPDH. As a comparison to latent infection, cultures of PBMC and enriched CD4+ T-cells from infected cats were activated with mitogens *ex vivo* [5], confirmed to be transcriptionally active (detectable FIV *gag* RNA and 2-LTR circle junction DNA), and then examined by ChIP. Positive controls were cultures of PBMC or CD4+ T-cells, obtained from healthy uninfected cats, which were inoculated with FIV *in vitro*; uninfected cells served as a negative control for the assay.

**Figure 3:**
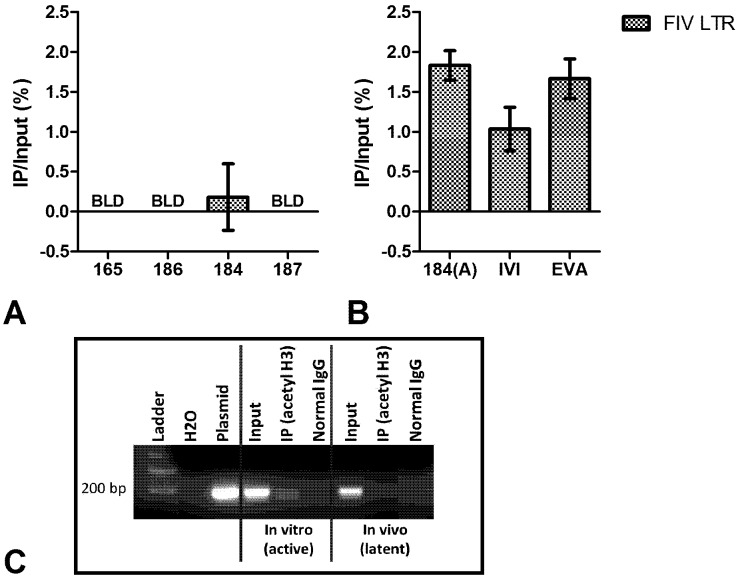
ChIP assay of FIV-infected PBMC using anti-acetylated histone H3 antibody. (**A**) Latently infected (based on undetectable FIV *gag* RNA) PBMC from three cats were below the limit of detection (BLD) for an association of the FIV LTR with acetylated H3, and detection of this complex for the fourth cat (184) was not significantly different from the background control; (**B**) Productively infected PBMC represented by a period of viral activation in monocytes (184(A)), *in vitro* infected (IVI) cells, or *ex vivo* activated (EVA) latently infected cells are all significantly associated with acetylated H3. Data are presented as the percentage of immunoprecipitated (IP) DNA out of the total input DNA (total amount of sheared chromatin DNA used for the assay) with background subtraction using a normal IgG control. Error bars represent the standard deviation of triplicate qPCR measurements, and data is representative of ChIP experiments repeated a minimum of two times between 16 and 29 months post FIV infection; (**C**) A representative gel image of the 180 bp FIV LTR products electrophoresed post real-time PCR for *in vitro* infected (transcriptionally active) and *in vivo*-derived (transcriptionally latent) FIV-infected PBMC. The immunoprecipitated (IP) product is detected in transcriptionally active, but not in latent, cell types.

**Figure 4:**
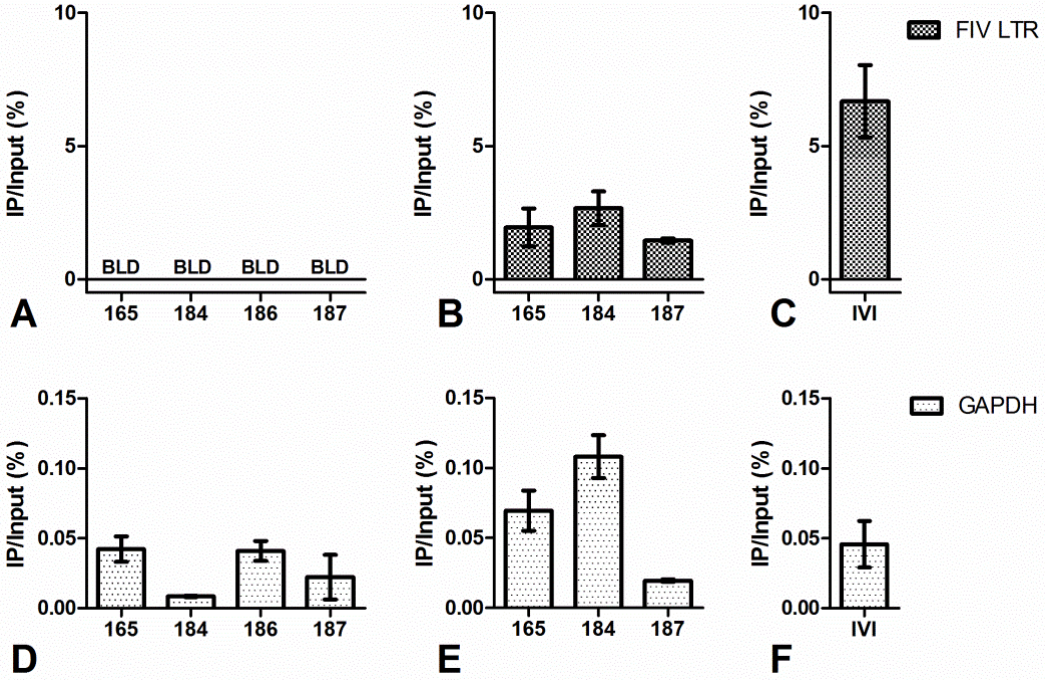
ChIP assay of FIV-infected CD4+ T-cells using anti-acetylated histone H3 antibody. (**A**) Latently infected (based on undetectable FIV gag RNA) CD4+ T-cells from all four chronically infected cats are below the limit of detection (BLD) for association of the FIV LTR with acetylated H3; (**B**) *Ex vivo* activated, productively infected CD4+ T-cells from 3 FIV-infected cats are all associated with acetylated H3; (**C**) *In vitro* infected SPF feline CD4+ T-cells are also associated with acetylated H3; (**D,E,F**) GAPDH positive controls are shown for data in A, B, and C respectively. Data are presented as the percentage of immunoprecipitated (IP) DNA out of the total input DNA with background subtraction using a normal IgG control. Error bars represent the standard deviation of triplicate qPCR measurements, and experiments were performed between 24 and 32 months post FIV infection.

**Figure 5:**
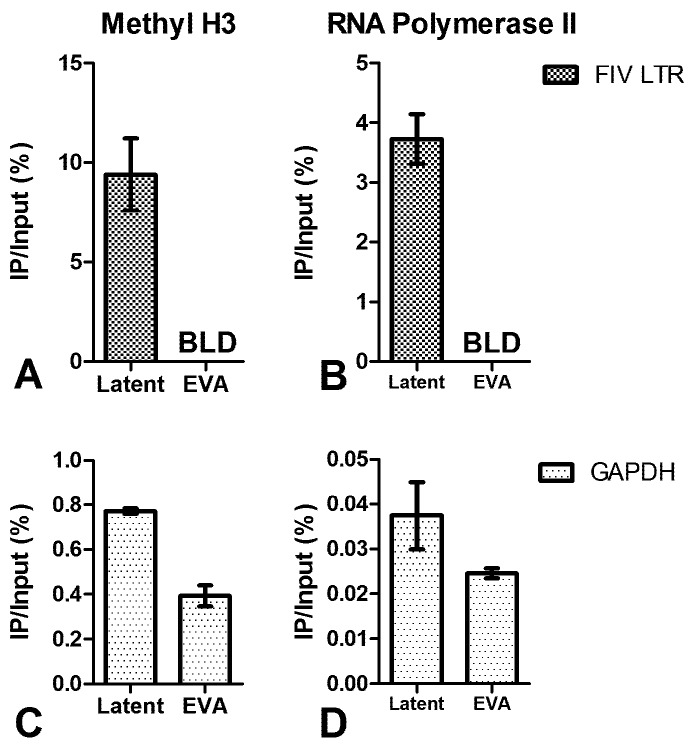
ChIP assay of FIV-infected CD4+ T-cells using anti-methylated histone H3 and anti-RNA Polymerase II antibodies. (**A**) The FIV promoter of latently-infected, but not *ex vivo* activated (EVA) [5], CD4+ T-cells is associated with methylated H3; (**B**) The FIV promoter of latently-infected, but not *ex vivo* activated (EVA), CD4+ T-cells is associated with RNA Polymerase II; (**C,D**) GAPDH positive controls are shown for A and B respectively. Data are presented as the percentage of immunoprecipitated (IP) DNA out of the total input DNA with background subtraction using a normal IgG control. Error bars represent the standard deviation of triplicate qPCR measurements, and data is representative of ChIP experiments repeated in two different cats (A/C: cat #184 and #187, B/D: cat #165 and #187) between 29 and 32 months post FIV infection.

ChIP analysis showed that the viral promoter in PBMC (Figure 3a) and peripheral CD4+ T-cells from FIV-infected cats (Figure 4a) was not associated with acetylated H3. However, an association with acetylated H3 was readily detected in the ChIP analysis of the viral promoter in cells activated *ex vivo* with mitogens or infected *in vitro* (Figure 3b, Figure 4b-c). This relationship remained consistent when immunoprecipitated (IP) DNA from transcriptionally active cells was diluted to a level which enabled normalization of the input FIV promoter DNA from both active and inactive cell types (data not shown). After amplification by real-time PCR, amplicons were electrophoresed on agarose gel to visualize the IP band in active cell types (Figure 3c). This band was purified from the gel, sequenced, and determined to be identical to the consensus sequence of inoculating virus (data not shown). Uninfected cats were negative for FIV LTR sequences in all DNA isolated from ChIP experiments. In CD4+ T-cells, the latent FIV promoter was associated with both methylated H3 (Figure 5a) and RNA polymerase II (Figure 5b); this association was not detected in transcriptionally active cells. These data suggest that the local chromatin environment of the *in vivo*-derived transcriptionally latent FIV promoter is repressive in nature (histone deacetylation and methylation), which may contribute to the transcriptional inactivity of the viral promoter. Furthermore, as demonstrated *in vitro* for HIV [12,13], the data indicate that RNA polymerase II remains paused on the FIV LTR.

## 4. Short Transcripts

To characterize the FIV transcriptional profile during latency, RNA was isolated using TRIzol (Invitrogen) from freshly isolated, latently infected (as defined by lack of viral *gag* mRNA) CD4+ T-cells and assayed for promoter-proximal short FIV transcripts by real-time PCR. Such short transcripts are consistent with pausing of the RNA polymerase complex [14]. Primer sets for FIV *gag* capsid, a 66-nucleotide region of R (+1 to +66), a 118-nucleotide region of R-U5 (+1 to +118), and an 87-nucleotide region at the 3’ terminus of the *orfA* gene were used. Only the short R fragment could be detected in freshly-isolated, latent CD4+ T-cells (Table 2). Copy number was normalized to GAPDH cDNA, and all viral transcripts (*gag*, R, R-U5, *orfA*) were detected in cells with active FIV replication. These data suggest that RNA polymerase II paused on the FIV promoter has limited processivity, as observed in PBMC from individuals infected with HIV [15], thereby transcribing a short R segment, but not extending into the viral *gag* gene (necessary for viral replication). This is particularly intriguing given that FIV is not known to encode a potent transcriptional transactivator (like HIV Tat) [16,17], and may imply that the promoter-associated repressive chromatin structure itself is highly relevant to latency.

**Table 2:** Real-time PCR amplifications of cDNA isolated from FIV-infected CD4+ T-cells.

Cat Number	Gag Copies	R Copies	R-U5 Copies	OrfA Copies
165	BLD	16,000 (±2,000)	BLD	BLD
184	BLD	30,000 (±20,000)	BLD	BLD
186	BLD	3,900 (±900)	BLD	BLD
187	BLD	20,000 (±10,000)	BLD	BLD
184(A)	1,600 (±100)	690,000 (±60,000)	1,200 (±100)	3,100 (±300)

Data are presented as the mean (± standard deviation) of triplicate PCR measurements per 10^6^ copies feline GAPDH cDNA, and are representative of CD4+ T-cell isolation at two different time points (30–34 months post FIV infection) for each cat. Sample labeled 184(A) represents PBMC from cat 184 during a period of viral activation in blood monocytes; BLD indicates values below the limit of detection.

## 5. Conclusions

In summary, this study demonstrated similarity in T-cell proviral loads and infectious virus between FIV-infected cats and HIV-infected individuals, as well as parallels between the *in vivo* transcriptional status of each of these viruses in their respective hosts’ CD4+ T-cells. Importantly, we implicated chromatin remodeling in the regulation of the latent FIV promoter *in vivo*; similarly, *in vitro* studies support the role of post-translational modifications of histones in the control of HIV-1 transcription [10]. During highly active antiretroviral therapy (HAART), HIV persists in infected individuals as a transcriptionally inactive (latent) integrated provirus [18], and memory CD4+ T-cells are the primary long-lived lentiviral reservoir [19,20,21]. Multiple molecular mechanisms may underlie the establishment and maintenance of latently infected cellular reservoirs [10,22], including epigenetic modification of histone proteins in chromatin [23,24,25]. Accordingly, FIV-infected cats provide an *in vivo* model of lentiviral latency in its natural host and may help to address questions that are either logistically or ethically not feasible to explore in HIV-infected humans, including the investigation of novel anti-latency therapeutic approaches [26].

## References

[1] Kanzaki L.I., Looney D.J. (2004). Feline immunodeficiency virus: A concise review. Front. Biosci..

[2] Burkhard M.J., Dean G.A. (2003). Transmission and immunopathogenesis of fiv in cats as a model for hiv. Curr. HIV Res..

[3] Elder J.H., Lin Y.C., Fink E., Grant C.K. (2010). Feline immunodeficiency virus (fiv) as a model for study of lentivirus infections: Parallels with hiv. Curr. HIV Res..

[4] Diehl L.J., Mathiason-Dubard C.K., O’Neil L.L., Obert L.A., Hoover E.A. (1995). Induction of accelerated feline immunodeficiency virus disease by acute-phase virus passage. J. Virol..

[5] Murphy B., Vapniarsky N., Hillman C., Castillo D., McDonnel S., Moore P., Luciw P.A., Sparger E.E. (2012). Fiv establishes a latent infection in feline peripheral blood cd4+ t lymphocytes *in vivo* during the asymptomatic phase of infection. Retrovirology.

[6] Chun T.W., Carruth L., Finzi D., Shen X., DiGiuseppe J.A., Taylor H., Hermankova M., Chadwick K., Margolick J., Quinn T.C. (1997). Quantification of latent tissue reservoirs and total body viral load in hiv-1 infection. Nature.

[7] Josefsson L., King M.S., Makitalo B., Brannstrom J., Shao W., Maldarelli F., Kearney M.F., Hu W.S., Chen J., Gaines H. (2011). Majority of cd4+ t cells from peripheral blood of hiv-1-infected individuals contain only one hiv DNA molecule. Proc. Natl. Acad. Sci. USA.

[8] Pedersen N.C., Leutenegger C.M., Woo J., Higgins J. (2001). Virulence differences between two field isolates of feline immunodeficiency virus (fiv-apetaluma and fiv-cpgammar) in young adult specific pathogen free cats. Vet. Immunol. Immunopathol..

[9] Pace M.J., Agosto L., Graf E.H., O’Doherty U. (2011). Hiv reservoirs and latency models. Virology.

[10] Colin L., van Lint C. (2009). Molecular control of hiv-1 postintegration latency: Implications for the development of new therapeutic strategies. Retrovirology.

[11] Liu G., Xia T., Chen X. (2003). The activation domains, the proline-rich domain, and the c-terminal basic domain in p53 are necessary for acetylation of histones on the proximal p21 promoter and interaction with p300/creb-binding protei. J. Biol. Chem..

[12] Zhang Z., Klatt A., Gilmour D.S., Henderson A.J. (2007). Negative elongation factor nelf represses human immunodeficiency virus transcription by pausing the rna polymerase II complex. J. Biol. Chem..

[13] Lenasi T., Contreras X., Peterlin B.M. (2008). Transcriptional interference antagonizes proviral gene expression to promote hiv latency. Cell Host Microbe.

[14] Peterlin B.M., Price D.H. (2006). Controlling the elongation phase of transcription with p-tefb. Mol. Cell.

[15] Adams M., Sharmeen L., Kimpton J., Romeo J.M., Garcia J.V., Peterlin B.M., Groudine M., Emerman M. (1994). Cellular latency in human immunodeficiency virus-infected individuals with high cd4 levels can be detected by the presence of promoter-proximal transcripts. Proc. Natl. Acad. Sci. USA.

[16] Chatterji U., de Parseval A., Elder J.H. (2002). Feline immunodeficiency virus orfa is distinct from other lentivirus transactivators. J. Virol..

[17] Gemeniano M.C., Sawai E.T., Leutenegger C.M., Sparger E.E. (2003). Feline immunodeficiency virus orf-ais required for virus particle formation and virus infectivity. J. Virol..

[18] Trono D., Van Lint C., Rouzioux C., Verdin E., Barre-Sinoussi F., Chun T.W., Chomont N. (2010). Hiv persistence and the prospect of long-term drug-free remissions for hiv-infected individuals. Science.

[19] Chomont N., El-Far M., Ancuta P., Trautmann L., Procopio F.A., Yassine-Diab B., Boucher G., Boulassel M.R., Ghattas G., Brenchley J.M. (2009). Hiv reservoir size and persistence are driven by t cell survival and homeostatic proliferation. Nat. Med..

[20] Blankson J.N., Persaud D., Siliciano R.F. (2002). The challenge of viral reservoirs in hiv-1 infection. Annu. Rev. Med..

[21] Chun T.W., Stuyver L., Mizell S.B., Ehler L.A., Mican J.A., Baseler M., Lloyd A.L., Nowak M.A., Fauci A.S. (1997). Presence of an inducible hiv-1 latent reservoir during highly active antiretroviral therapy. Proc. Natl. Acad. Sci. USA.

[22] Margolis D.M. (2010). Mechanisms of hiv latency: An emerging picture of complexity. Curr. HIV/AIDS Rep..

[23] Imai K., Togami H., Okamoto T. (2010). Involvement of histone h3 lysine 9 (h3k9) methyltransferase g9a in the maintenance of hiv-1 latency and its reactivation by bix01294. J. Biol. Chem..

[24] Margolis D.M. (2011). Histone deacetylase inhibitors and hiv latency. Curr. Opin. HIV AIDS.

[25] Friedman J., Cho W.K., Chu C.K., Keedy K.S., Archin N.M., Margolis D.M., Karn J. (2011). Epigenetic silencing of hiv-1 by the histone h3 lysine 27 methyltransferase enhancer of zeste 2. J. Virol..

[26] Geeraert L., Kraus G., Pomerantz R.J. (2008). Hide-and-seek: The challenge of viral persistence in hiv-1 infection. Annu. Rev. Med..

